# Role of TGFβ in regulation of the tumor microenvironment and drug delivery (Review)

**DOI:** 10.3892/ijo.2015.2816

**Published:** 2015-01-07

**Authors:** PANAGIOTIS PAPAGEORGIS, TRIANTAFYLLOS STYLIANOPOULOS

**Affiliations:** 1 Cancer Biophysics Laboratory, Department of Mechanical and Manufacturing Engineering, University of Cyprus, Nicosia 1678, Cyprus; 2 Department of Health Sciences, Program in Biological Sciences, European University Cyprus, Nicosia 1516, Cyprus

**Keywords:** cancer-associated fibroblasts, immune cells, desmoplasia, extracellular matrix, cancer therapy

## Abstract

Deregulation of cell signaling homeostasis is a predominant feature of cancer initiation and progression. Transforming growth factor β (TGFβ) is a pleiotropic cytokine, which regulates numerous biological processes of various tissues in an autocrine and paracrine manner. Aberrant activity of TGFβ signaling is well known to play dual roles in cancer, depending on tumor stage and cellular context. The crucial roles of TGFβ in modulating the tumor microenvironment, its contribution to the accumulation of mechanical forces within the solid constituents of a tumor and its effects on the effective delivery of drugs are also becoming increasingly clear. In this review, we discuss the latest advances in the efforts to unravel the effects of TGFβ signaling in various components of the tumor microenvironment and how these influence the generation of forces and the efficacy of drugs. We also report the implications of tumor mechanics in cancer therapy and the potential usage of anti-TGFβ agents to enhance drug delivery and augment existing therapeutic approaches. These findings provide new insights towards the significance of targeting TGFβ pathway to enhance personalized tumor treatment.

## 1. Introduction

The crucial role of transforming growth factor β (TGFβ) in tumor progression, metastasis and treatment has been well recognized and has become the topic of extensive research. Among the effects, TGFβ can regulate cancer cell proliferation, contribute to epithelial-to-mesenchymal transition (EMT), suppress the function of immune cells compromising immune response, contribute to the conversion of fibroblasts to myofibroblasts and cause overproduction of extracellular matrix (ECM) in the tumor. While it has been known for over two decades that anti-cancer drugs cannot penetrate deep into collagen-rich tumors (e.g., pancreatic cancers) and, more significantly, that depletion of collagen fibers can improve drug delivery, only recently TGFβ has become a target to reduce tumor fibrosis and thus, increase intratumoral drug concentration and treatment efficacy. Preclinical data of this new strategy are promising and it has already reached clinical trials. In this review, we first present a brief description of TGFβ synthesis and activation along with its signaling pathways. Following, we discuss the effects of TGFβ on tumor progression, its pathway alterations in cancer as well as its effects on EMT, immune cells function, fibroblasts behavior and ECM remodeling. Finally, based on the above, we review the barriers to the effective delivery of drugs caused by TGFβ and how regulation of TGFβ signaling can be employed to optimize delivery of therapeutic agents and overall survival (1–3).

## 2. TGFβ synthesis and activation

**Figure 1:**
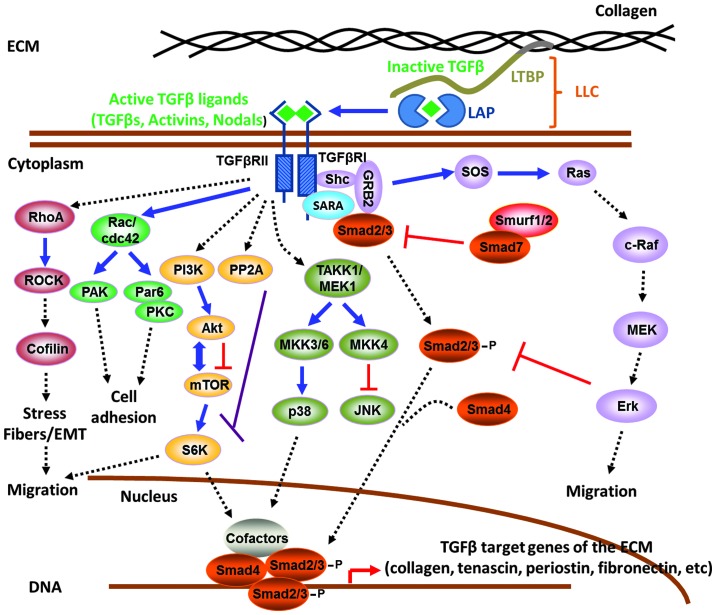
Smad-dependent and -independent transforming growth factor β (TGFβ) pathways. TGFβ is initially synthesized and stored in the extracellular matrix (ECM) in an inactive form, as part of the large latent complex (LLC). Upon activation, the released TGFβ ligands initiate signaling by binding to TGFβRIs and TGFβRIIs. TGFβ receptors (TGFβRs) exhibit kinase activities that are necessary for transducing canonical TGFβ signaling by phosphorylating Smads2/3. Activated receptor-associated Smads can form a heterotrimeric complex with Smad4, which interacts with other co-factors in the nucleus to regulate the expression of *TGFβ* target genes. In addition, downstream intracellular signaling may also be transduced via auxiliary pathways including the MEK/Erk, the Rho-like GTPases, the phosphatidylinositol-4,5-bisphosphate 3-kinase/Akt (PI3K/Akt) and the p38/mitogen-activated protein kinase (MAPK) pathways to regulate biological responses such as epithelial-to-mesenchymal transition (EMT), cell adhesion, migration and survival.

The TGFβ superfamily encompasses around 40 secreted cytokines, including TGFβ, bone morphogenetic proteins (BMPs), activins, nodal, lefty, myostatin, anti-Müllerian hormone (AMH) and growth differentiation factors (GDFs). These cytokines regulate a plethora of biological functions such as cell proliferation and apoptosis, embryonic patterning, stem cell maintenance, cell differentiation, migration and immune surveillance. Importantly, the effects of these factors are characterized as cell-type specific as well as context dependent (1–3). The TGFβ isoforms, with most common being TGFβ1, 2 and 3, are initially synthesized as 75 kDa inactive homodimers, known as pro-TGFβ, which consist of TGFβ associated with latency-associated proteins (LAPs) at the N-terminal part of the pro-peptide. This is part of the TGFβ large latent complex (LLC), comprised of the LAPs and the latency TGFβ-binding proteins (LTBPs) (4–7), and is covalently associated to the ECM via the N-terminal region of LTBPs (8,9) (Fig. 1). While TGFβ is part of the LLC complex, it remains in an inactive form since the high affinity association of LAPs with TGFβ prevents the interaction with its receptors (10). During TGFβ activation, LAPs undergo conformational changes induced by thrombospondin-1 (TSP-1) (11,12) followed by cleavage mediated by furin convertase, plasmin or matrix metalloproteinases MMP-2/9 resulting in the release of the mature 24 kDa TGFβ dimer (13–15). The active ligand is then able to bind and activate TGFβ receptors (TGFβRs) to propagate downstream intracellular signaling events. Therefore, the processing of pro-TGFβ into the active TGFβ ligand is a critical regulatory step which determines its bioavailability.

## 3. TGFβ signaling pathways

The TGFβ and TGFβ-like cytokines mediate downstream intracellular signaling via the Smad family of proteins, which consists of eight human structurally related members (16–20) (Fig. 1). Smads can be functionally classified into three groups: the receptor activated Smads (R-Smads), which include Smad1, 2, 3, 5, 8; the common mediator Smad (Co-Smad), Smad4; and the inhibitory Smads (I-Smads), Smad6 and 7 (17,21). Three types of TGFβRs are responsible for initiating signaling; TGFβRI, II and III. There are seven TGFβRI, five TGFβRII and two TGFβRIII known so far. TGFβRIs include activin receptor-like kinases 1–7 (ALK1–7), TGFβRIIs include the TGFβRII, bone morphogenetic protein receptor II (BMPRII), activin receptor II (ACTRII), ACTRIIB, anti-Müllerian hormone receptor II (AMHRII), while beta-gycan and endoglin belong to the TGFβRIIIs (22) and mostly function as co-receptors to enhance activin signaling (23). In most tissues, TGFβ ligands function through heteromeric complex formation between two TGFβRI and two TGFβRII molecules. While both receptors possess Ser/Thr kinase activity, TGFβRIIs function as the ‘activator’ and TGFβRIs as the ‘signal propagating’ component (24). The TGFβRII-ALK5 complex transduces the signal from all three TGFβ isoforms in multiple cell types, whereas association of TGFβRII with ALK1 is involved in endothelial cells and with ALK2 in cardiovascular tissues (25). ALK5 activates Smad2 and 3 via the canonical TGFβ signaling pathway whereas ALK2, 3 and 6 can activate Smad1, 5 and 8, which are transducers of the BMP signaling pathway (26,27). The TGFβ signaling pathways can be classified in two major categories; the canonical or Smad-dependent and the non-canonical or Smad-independent pathways.

### Canonical pathway (Smad-dependent)

Even though TGFβ isoforms may elicit diverse cellular responses, they all activate signaling via a similar sequence of events. Binding of the active TGFβ1 ligand to the Ser/Thr kinase TGFβRII followed by recruitment of the ALK5 (TGFβRI) on the cell surface initiates intracellular signaling. Within the heterotetrameric receptor-ligand complex formed, TGFβRII phosphorylates TGFβRI allowing it to interact with the R-Smads (Smad2/3) which, in turn, become phosphorylated at the conserved SSXS C-terminal motif (28,29). Recruitment of R-Smads to the activated TGFβRI is facilitated by Smad anchor for receptor activation (SARA) protein (30). Subsequently, this triggers the formation of a heterotrimeric complex between phosphorylated R-Smads (Smad2/3) and Co-Smad (Smad4), which can translocate into the nucleus to regulate gene expression (3) (Fig. 1). Smads can differentially modulate gene expression by acting as transcription factors in co-operation with co-activators, such as p300/CREB-binding protein (CBP), p300/CBP-associated factor (PCAF), Smad4-interacting factor (SMIF), forkhead transcription factors 1, 3, 4 (FoxO1/3/4), specificity protein 1 (Sp1), c-Jun/c-Fos, Sertad1, or co-repressors, such as E2F4/5-p107, activating transcription factor 3 (ATF3), TGFβ-induced factor (TGIF), Ski, SnoN, forkhead transcription factor G1 (FoxG1), ecotropic viral integration site 1 protein (EVI1) and C-terminal binding protein (CTBP) (28,31–47). In addition, Smads are able to epigenetically regulate gene expression either by inducing chromatin remodeling (48,49) or by maintaining DNA methylation and silencing of selected genes (50). Importantly, the I-Smad, *Smad7*, is a key target gene induced by TGFβ signaling and acts as negative feedback regulator of the pathway (51). In the absence of TGFβ stimulation, Smad7 resides in the cell nucleus and translocates to the plasma membrane upon TGFβ-mediated receptor activation (52). Smad7 is then able to interfere and block interactions between the R-Smads and the activated receptors to inhibit downstream signaling events (53). In addition, Smad7 can target the TGFβRs for proteasomal degradation via the E3-ubiquitin ligases Smurf1 and 2 (54,55). Finally, Smad7 antagonizes the formation of a functional Smad-DNA complex by directly binding to DNA via its MH2 domain and therefore blocks TGFβ-mediated transcriptional responses (56).

### Non-canonical pathways (Smad-independent)

It is also well established that TGFβ-mediated effects can also be exerted through non-canonical Smad-independent pathways (57). TGFβ has been shown to induce activation of Erk signaling in various tissues including epithelial and endothelial cells, fibroblasts, breast and colorectal cancer cells in order to promote disassembly of adherens junctions and cell migration (58–64). TGFβRI phosphorylation can recruit and activate ShcA, thus promoting the formation of a ShcA/Grb2/Sos complex. In turn, this complex is able to activate Ras on the plasma membrane followed by sequential activation of c-Raf, MEK and Erk (65).

Moreover, TGFβ can mediate the activation of the c-Jun N-terminal kinase (JNK) and p38/mitogen-activated protein kinase (MAPK) pathways, which are responsible for promoting apoptosis or cell migration depending on cellular context (66–68), via the mitogen-activated protein kinase kinase (MKK)4 and 3/6, respectively (69,70). Further upstream, MKKs are phosphorylated by the TGFβ-activated kinase 1 (TAK1) (71,72) which is recruited to the TGFβRs via the scaffold protein TNF receptor-associated factor 6 (TRAF6) (73,74). Besides TAK1, two other mitogen-activated protein kinase kinase kinases (MAPKKKs), namely MEKK1 and mixed lineage kinase 3 (MLK3), were also shown to mediate TGFβ-induced activation of JNK and p38-MAPK by MKK4 and 3/6 (75,76).

The Rho-like small GTPases, predominantly RhoA, Rac and cell division cycle 42 (cdc42), are additional molecules that mediate important TGFβ cellular functions, such as cytoskeletal organization, cell polarity, cell migration and gene expression (77). TGFβ is able to rapidly activate the RhoA and cdc42/Rac1 pathways, in a Smad2/3-independent manner, to promote actin polymerization, formation of stress fibers and EMT (78,79). TGFβ may also downregulate RhoA protein levels by recruitment of Par6 at the TGFβRI–II complex. Phosphorylation of Par6 by TGFβRII triggers binding of the E3 ligase Smurf1 to the complex followed by ubiquitination and degradation of RhoA at sites of cellular protrusions. Subsequently, this leads to the dissolution of tight junctions, rearrangement of actin cytoskeleton and EMT (80).

Some of the effects exerted by TGFβ could also be mediated by activation of the phosphatidylinositol-4,5-bisphosphate 3-kinase/Akt (PI3K/Akt) pathway. This is evident from studies showing that TGFβ can rapidly induce PI3K activation followed by phosphorylation of its effector Akt to promote EMT, cell migration and survival (81,82). One of the most important effector molecules downstream of PI3K/Akt pathway appears to be the mammalian target of rapamycin (mTOR), a key regulator of protein synthesis, which can subsequently phosphorylate S6 kinase (S6K) and eukaryotic initiation factor 4E-binding protein 1 (4EBP1) (83). Activation of the mTOR pathway by TGFβ is thought to be important for regulating cell size, EMT and invasion (84) (Fig. 1).

## 4. TGFβ signaling in cancer initiation and tumor progression

It is well established that the multipotent actions of TGFβ are highly context dependent. The complexity of these functions is increased due to the fact that TGFβ exerts distinct effects depending on the tissue type as well as the genetic and epigenetic background of cells (85). It is clearly evident that TGFβ plays dual roles during carcinogenesis. In early stages TGFβ promotes growth inhibition and apoptosis of normal epithelial and lymphoid cells as well as pre-malignant tumors, whereas during late stages TGFβ acquires pro-oncogenic and pro-metastatic roles, which are associated with a progressive increase in the locally secreted TGFβ levels (86–88). Therefore, one of the hallmarks of cancer is that the vast majority of cases exhibits insensitivity to TGFβ-mediated growth inhibition.

### Regulation of cell proliferation

It has long been noted that TGFβ has a cytostatic effect on normal epithelial (89), endothelial (90,91) and neuronal cells (92) as well as certain cells of the immune system, such as T cells (93). These functions of TGFβ are extremely important for physiological tissue homeostasis in order to restrain cell proliferation and prevent the generation of hyperproliferative disorders, like cancer. These anti-proliferative effects primarily control the G1/S phase transition events (94) and are mediated via induction of the cyclin-dependent kinase inhibitors *CDKN2B* (encoding p15/INK4B) (95), *CDKN1A* (encoding p21/Cip/Waf1) (96) and p27/Kip1 (97) by TGFβ. Cell cycle arrest can also be achieved by repression of the proliferation-inducing transcription factors c-Myc (98) and the family of inhibitor of DNA-binding proteins ID1, 2 and 3 (36,99). On the other hand, the effects of TGFβ in proliferation can be opposing, depending on the tissue type. It is also well recognized that TGFβ enhances proliferation of fibroblasts (89) and it is often mediated indirectly by TGFβ-induced connective tissue growth factor (CTGF) secretion, which is responsible for stimulating fibroblast proliferation and ECM synthesis (100). It is now unambiguously accepted that cancer-associated fibroblasts (CAFs) play critically important roles in the tumor microenvironment and cancer progression and their functions are further discussed below.

### Pathway alterations in human cancers

Numerous human studies have identified that components of the TGFβ pathway become genetically or epigenetically altered in various tumor types thus explaining, at least in part, the escape from TGFβ-mediated growth control. Loss of function or truncating mutations in *TGFβRI* and *TGFβRII* as well as in *Smad2* and *Smad4* have been detected in colorectal, pancreatic, gastric and prostate cancers (18,101–105). In addition, loss of the 18q21 chromosome region, harboring the *Smad4* gene, is commonly observed in ~60% of pancreatic and 30% of colorectal cancers (106–109) has been shown to promote angiogenesis and tumor growth by inducing vascular endothelial growth factor (VEGF) expression (60,110). However, in other tumor types like breast, the frequency of *Smad* gene mutations is rare (18,104,105) suggesting that alternative mechanisms for acquiring resistance to growth inhibition by TGFβ exist. These include activation of the Ras oncogene which leads to Erk-mediated Smad2/3 phosphorylation and suppression of functional Smad complex formation (111–113). Furthermore, overexpression of the dominant-negative CCAAT/enhancer-binding protein β (C/EBPβ) isoform LIP in breast cancer patients was found to suppress TGFβ-mediated growth inhibition (114). Finally, another mechanism which TGFβ may exploit in order to switch from a tumor suppressor to a metastasis-promoting factor is through differential regulation of the *ID1* gene. While ID1 expression is suppressed by TGFβ in normal tissues, it was found to be induced in patient-derived metastatic breast cancer cells (115).

### EMT and cancer metastasis

EMT is an integral process during embryonic development which can be abnormally reactivated in adult tissues under pathological conditions, such as cancer and fibrosis (116). It involves the activation of a coordinated reversible transcriptional program whereby epithelial cells undergo dissolution of cell junctions, lose their polarity and epithelial characteristics concomitantly with acquisition of mesenchymal features and dramatic remodeling of their cytoskeleton. During this process, the expression of epithelial genes, such as *E-cadherin*, *γ-* and *β-catenin*, *zonula occludens* (*ZO*), and *claudins* is suppressed with concurrent expression of mesenchymal components, such as N-cadherin, vimentin, fibronectin and α-smooth muscle actin (α-SMA) (50,117,118). This program can be initiated by several pleiotropically acting transcription factors regulated by signaling pathways such as TGFβ, Wnt and receptor tyrosine kinases (RTKs). Some of the better characterized examples include Snail (119), Slug (120), zinc-finger E-box binding homeobox 1 (ZEB1/δEF1) (121), zinc-finger E-box binding homeobox 2/Smad interacting protein 1 (ZEB2/SIP1) (122), Twist (117), high mobility group AT-hook 2 (HMGA2) (123) and forkhead box protein C2 (FOXC2) (124). In addition, recent studies indicate that overactive TGFβ-TGFβR-Smad2 signaling axis could further contribute to the establishment of an EMT phenotype by maintaining the epigenetic silencing of epithelial genes during this process (50). Besides Smads, other signaling pathways have also been implicated in TGFβ-induced EMT, including Erk, PI3K/Akt, RhoA, p38-MAPK and cofilin (125–127). Induction of EMT is one of the major mechanisms by which TGFβ has been shown to promote cell motility, invasiveness and metastasis of cancer cells (128). EMT significantly enhances intravasation of carcinoma *in situ* cells through the basement membrane, survival in the circulation, extravasation at the distal tissues and formation of micrometastases in secondary organs (116,117,129).

## 5. The effects of TGFβ on the tumor microenvironment

Under physiological conditions, the sustained local release of basal TGFβ levels is sufficient to maintain normal tissue homeostasis. However, under conditions of tissue injury, the local TGFβ secretion from stromal cells and blood platelets is rapidly increased to facilitate wound repair as well as to prevent uncontrolled regenerative cell proliferation and inflammation (130,131). A similar situation is commonly observed in pre-malignant tumors where TGFβ is secreted in the microenvironment initially to control proliferation and cancer progression, but it is ultimately utilized by cancer cells to promote their malignant properties. Local TGFβ release produces a tumor microenvironment which is conducive to tumor growth, invasion and metastasis (132). Secretion of TGFβ can be derived from epithelial cancer cells thus regulating their own properties within the tumor mass in an autocrine or paracrine fashion (125). Moreover, infiltrating stromal cells, including fibroblasts, leukocytes, macrophages, bone-marrow derived endothelial, mesenchymal and myeloid precursor cells, is another major source of this cytokine (133). Finally, TGFβ can be stored in the ECM of the bone and can be activated during development of osteolytic metastatic lesions (134). In the following paragraphs, we summarize the effects of TGFβ on the main and better characterized components of the tumor microenvironment and particularly on fibroblasts, immune cells and the ECM.

### Effect of TGFβ on immune cells

TGFβ exhibits immunosuppressive effects on all arms of the immune system because it functions as antagonist of several functions of the immune cells (132,135). As a result, the anti-tumor immune response is compromised, reducing cancer cell recognition and clearance. Specifically, TGFβ affects the function of natural killer cells, CD4^+^ and 8^+^ T cells, macrophages, neutrophils, dendritic, mast and B cells (136–138). Specifically, a TGFβ-rich tumor microenvironment is a suppressor of T-cell proliferation, reduces their effector function and inhibits the maturation of T helper cells (137,139,140). It also induces macrophage M2 polarization from a type I to a type II phenotype, which hinders the suppression of monocyte-mediated cell death, reduces effector function and increases chemotaxis (141,142). Additionally, TGFβ induces an N2 neutrophil phenotype which, as with the macrophages, reduces effector function and increases secretion of inflammatory cytokines (143). Finally, high levels of TGFβ can cause apoptosis of B cells, inhibit the maturation of dendritic and natural killer cells and induce chemotaxis of mast cells (144–146). The combined immuno-suppressive effects of TGFβ compromise the ability of the host to resist tumor progression and thus consist a barrier to immunotherapy.

### Effect of TGFβ on fibroblasts

A primary role of TGFβ in modulating the tumor microenvironment is its contribution to the conversion of fibroblasts to myofibroblasts, also known as CAFs (147,148). Specifically, the compressive forces developed inside a tumor, due to its growth in the confined space of the host tissue, can facilitate the conversion of fibroblasts to proto-myofibroblasts. Subsequently, TGFβ increases the levels of collagens I and III and fibronectin, which promote cellular adhesion to extracellular fibers, and thus, enhances the communication of mechanical signals between the ECM of the tumor and the fibroblasts (149,150). As a result, the mechanical forces are more actively transmitted in the interior of the cell and contribute to the conversion of proto-myofibroblasts to differentiated myofibroblasts. Myofibroblasts are characterized by more extensively developed stress fibers in the cytoskeleton compared to proto-myofibroblasts, presumably to balance the extracellular forces, and by the *de novo* expression of α-SMA. The contraction of myofibroblasts is sustained by α-SMA stress fibers and it is regulated by Rho/ROCK signaling activation. The produced contractile forces remodel the ECM due to the ability of fibroblasts to stretch collagen fibers and produce ECM molecules (151,152). Additionally, these forces can be transmitted to the LLC via integrins. LLC is also bound to extracellular fibers (Fig. 1), which resists the pulling of the LLC by myofibroblasts and gives rise to a mechanically-induced liberation of TGFβ (147). The stiffer the ECM, the stronger the interactions among myofibroblasts, LLC and extracellular fibers and thus, the release of TGFβ becomes more pronounced. Therefore, myofibroblast contraction within a collagen-rich, and thus, stiff microenvironment further stimulates the release of active TGFβ from its latent form.

### Effect of TGFβ on ECM

TGFβ upregulates the expression and synthesis of many matrix proteins, primarily through the recruitment of myofibroblast. Proteins upregulated by TGFβ include collagens I–V, basement membrane proteins (laminin, entactin, perlecan) and ECM proteins (fibronectin, osteopontin, thrombospontin, tenascin, osteonectin/SPARC, elastin, biglycan, decorin, and hyaluronan) (153). Additionally, in the early stages of carcinogenesis, TGFβ stimulates myofibroblasts and other stromal cells to enhance the synthesis of collagen crosslinking enzymes, particularly lysyl oxidase, which increases the rigidity of the collagen network (154). On the contrary, TGFβ downregulates the synthesis of matrix-depleting proteins, such as matrix metalloproteinases (MMP-1, -8, -13). As a result, the increase in matrix protein synthesis and decrease in matrix proteinase activity, owing to the TGFβ activity, contributes to the remodeling of the tumor ECM and can result in a fibrotic response, known as desmoplasia, which is commonly observed in many types of tumors and particularly in pancreatic, colon and breast cancers as well as in various sarcomas (155,156).

**Figure 2:**
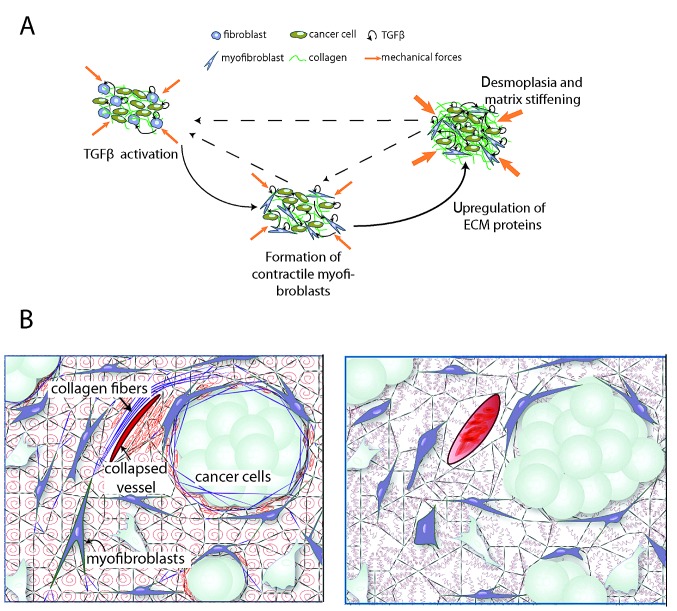
Role of mechanical forces and transforming growth factor β (TGFβ) in tumor desmoplasia and vessel compression. (A) TGFβ and mechanical forces contribute to the conversion of fibroblasts to contractile myofibroblasts. Myofibroblast formation results in upregulation of extracellular matrix (ECM) proteins and leads to tumor fibrosis, matrix stiffening and desmoplasia. Increased stiffening of the matrix, in turn, increases the magnitude of the mechanical forces and contributes to further activation of TGFβ from the ECM. This creates a positive feedback loop, which gives rise to a continuous activation of TGFβ and formation of myofibroblasts. (B) Upregulation of ECM proteins and the resulting increase in mechanical forces can compress and eventually collapse intratumoral blood vessels. Alleviation of these forces with an anti-TGFβ agent has the potential to decompress vessels and thus, improve perfusion and drug delivery to solid tumors [adapted with permission from (157)].

Tumor fibrotic response stiffens the tumor tissue, and as a result, it increases the compressive physical forces in the interior of the tumor (157). Compression of cancer cells alters their gene expression profile to enhance their invasive and metastatic phenotype (158,159). Furthermore, as mentioned previously, matrix stiffening along with the high contractile forces of myofibroblasts, cause further liberation of TGFβ from the LLC. These events suggest a positive feedback loop between TGFβ activation, myofibroblast contraction and ECM remodeling and production (Fig. 2A) (148). Finally, compression of intratumoral blood vessels reduces tumor perfusion, and thus, the delivery of oxygen (160). Hypo-perfusion and hypoxia, in turn contribute to immune-evasion, promote malignant progression and metastasis, and reduce the efficacy of a number of therapies including radiation treatment and systemic administration of chemo- and nanotherapy (161–163).

## 6. TGFβ, tumor desmoplasia and barriers to drug delivery

The desmoplastic reaction of solid tumors hinders all three transport steps of the systemic delivery of drugs, namely vascular, transvascular and interstitial transport (156,163). As mentioned above, increased levels of collagen in the ECM, result in intratumoral blood vessel compression and hypo-perfusion. Hypo-perfusion, in turn, reduces the concentration of the drug that can reach the tumor site. Apart from compromised drug delivery, hypo-perfusion also decreases the supply of oxygen rendering the tumor hypoxic, which in turn reduces the efficacy of radiation therapy. Additionally, desmoplasia reduces the hydraulic conductivity of the tumor interstitial space, i.e., the ease with which the interstitial fluid percolates through the interstitial space of a tissue. High hydraulic conductivity allows fluid to rapidly flow in the interstitial space and be drained by peripheral lymphatic vessels. The accumulation of collagen and other ECM proteins in tumors decrease the available spaces for interstitial fluid flow and because the fluid cannot freely move, the interstitial fluid pressure (IFP) increases. Interstitial hypertension is a hallmark of tumor pathophysiology. IFP reaches and even exceeds micro-vascular fluid pressure, which eliminates pressure gradients across the tumor vessel wall and thus, the transvascular transport of drugs (164). Therefore, the only mechanism of transport is through diffusion (i.e., due to a concentration difference), which is inversely proportional to the size of the therapeutic agent. Chemotherapeutic agents, with a size <1 nm, are able to diffuse fast and exit the tumor vasculature. Nanoparticles, however, with sizes >60 nm cannot effectively extravasate into the tumor interstitial space (165).

Furthermore, the dense interstitial matrix of desmoplastic tumors hinders the homogeneous distribution of large nanoparticles. As with transvascular transport, nanoparticles with a size >60 nm often cannot penetrate deep into the tumor because their size is comparable to the size of the pores of the interstitial collagen network and they often get trapped (166). Therefore, even if large nanoparticles extravassate from the leaky vessels of the tumor, they will not be able to effectively diffuse into the tissue but they will concentrate in the perivascular regions, causing only local effects. Apart from these steric interactions between the interstitial matrix and nanoparticles, the increased levels of collagen and hyaluronan give rise to electrostatic interactions. Indeed, hyaluronan has a highly negative charge, while collagen fibers carry a slight positive charge. Nanoparticles of a non-neutral surface charge density can be attracted electrostatically and bind to these proteins, which further inhibits their uniform delivery inside the tumor (167).

## 7. Therapeutic applications of TGFβ targeting

Pharmacological inhibition of TGFβ has been used in preclinical and clinical studies as a therapeutic strategy to either hinder tumor progression directly or modify the tumor micro-environment in order to improve perfusion and drug delivery and thus, increase indirectly the efficacy of the treatment. There is a large number of TGFβ inhibitory drugs employed in these studies (137). Particularly, targeting with TGFβ agents (e.g., 1D11, AP12009, SD-208) as well as non-specific targeting with other TGFβ inhibitory drugs (e.g., tranilast) have shown to reduce tumor progression and metastasis *in vivo*, mainly owing to augmentation of the immune response and inhibition of EMT (132,168–171). However, there are also studies that relate inhibition of TGFβ with promotion of tumor progression owing to an increase in inflammatory cell infiltration (172). Particularly, it has been shown that inflammatory infiltrates mediate the pro-tumorigenic functions of fibroblasts that lack TGFβ signalling. Clinical trials for the use of TGFβ inhibitory drugs have been in progress (ClinicalTrials.gov identifiers: NCT00368082, NCT01582269 and NCT00844064), but their results are not conclusive yet, presumably owing to differences in the degree of desmoplasia among tumor types or even among tumors of the same type, but also owing to the various effects of TGFβ on tumor biology.

Targeting of TGFβ to reduce desmoplasia has the ability to alleviate physical forces in tumors, decompress tumor blood vessels and improve perfusion (Fig. 2B) (160). Restoration of tumor perfusion, however, can increase nutrients supply to the tumor, and thus, increase its growth rate. Also, the decompressed vessels could allow more metastatic cells to leave the primary tumor. Indeed, in some cases, inhibition of TGFβ has been shown to facilitate tumor progression and metastases in mouse tumor models (173,174), whereas other studies, not related to TGFβ, have shown a correlation between improved perfusion and increased metastases (175,176). Therefore, based on this rationale, judicious doses of TGFβ inhibitory drugs should be used to alleviate physical forces, decompress blood vessels and improve perfusion when these agents are combined with cytotoxic treatments, such as chemo-, nano-, immuno- and radiotherapy. In these combined treatments the role of the anti-TGFβ drug is to enhance the delivery of the cytotoxic agent and thus, optimize its efficacy. This therapeutic strategy is known as stress alleviation treatment (156,163,165).

Detailed *in vivo* studies have shown that re-purposing the anti-hypertensive, angiotensin receptor blocker (ARB) drug losartan reduced expression of TGFβ1 and decreased stromal collagen and hyaluronan production, in doses that did not affect blood pressure. Reduction of collagen and hyaluronan, in turn, reduced stress levels in the tumor decompressing intratumoral blood vessels and improving perfusion. Furthermore, reduction of the ECM components improved the interstitial fluid flow and thus, reduced levels of IFP. Improved perfusion and reduced IFP enhanced the delivery and efficacy of chemotherapy in orthotopic breast and pancreatic murine tumor models (160). Also, in another study combined treatment of mice bearing tumors with losartan and nanomedicine (Doxil) increased the distribution of the drug and the overall survival of the mice (177). Furthermore, retrospective analyses of clinical data have shown increased survival in patients with lung or renal cancers treated with ARBs (178,179). Similar retrospective analysis has shown that patients with pancreatic ductal adenocarcinomas (PDACs) receiving ARBs survived ~6 months longer than those who did not (180). These preclinical and clinical data have led to a phase II clinical trial with losartan and FOLFIRINOX in PDAC patients (ClinicalTrials.gov identifier NCT01821729). Apart from the use of ARBs, the TGFβ neutralizing antibody 1D11 improved the distribution and efficacy of therapeutics in breast carcinomas by reducing the tumor stroma (181). Additionally, re-purposing the drug pirfenidone, a TGFβ inhibitor clinically approved for the treatment of idiopathic pulmonary fibrosis, was shown to suppress desmoplasia in mice bearing pancreatic tumors and improve the efficacy of chemotherapy (182). Apart from chemotherapy, radiation therapy has been also improved after treatment with TGFβ inhibitors. Efficacy of radiotherapy depends on the oxygenation of the tissue, which is regulated by tumor perfusion (183,184).

## 8. Conclusions and future perspectives

Owing to the pleiotropic effects of TGFβ on tumor microenvironment and progression, targeting TGFβ signaling to directly treat tumor growth remains controversial. Recent studies have suggested an alternative therapeutic strategy, which involves the use of anti-TGFβ agents in a stress alleviation treatment. The scope of this strategy is to hinder but not completely inhibit the activation of TGFβ ultimately aiming to reduce tumor desmoplasia and particularly the levels of collagen. As described in this review, reduced collagen levels can lead to improved delivery of both chemo- and nano-therapeutics by alleviating mechanical forces and decompressing intratumoral blood vessels. Thus, blocking of TGFβ can improve indirectly the efficacy of conventional treatments. It is promising that many anti-TGFβ agents exist that are already clinically approved for other diseases (e.g., ARBs for hypertension). Re-purposing of these drugs can lead to more effective anti-cancer therapies. Therefore, we need to identify safe and well-tolerated pharmaceutical agents that may complement the treatment regimen of cancer patients. Anti-TGFβ agents are not the only drugs that have the ability to modify the tumor microenvironment. In principle, any clinically approved agent that has the ability to reduce collagen levels could be employed as an alternative strategy. Also, collagen is not the only target for the stress alleviation treatment. Reduction of stromal cells or hyaluronan has also the potential to enhance drug delivery through the same mechanism (157).
